# The Impacts of Armed Conflicts and Civilian Uprisings on Children’s Health

**DOI:** 10.3390/children9121913

**Published:** 2022-12-07

**Authors:** Amir Khorram-Manesh

**Affiliations:** 1 Department of Surgery, Institute of Clinical Sciences, Sahlgrenska Academy, Gothenburg University, 41345 Gothenburg, Sweden; amir.khorram-manesh@surgery.gu.se; Tel.: +46-70-772-2741; 2 Gothenburg Emergency Medicine Research Group (GEMREG), Sahlgrenska Academy, Gothenburg University, 41305 Gothenburg, Sweden; 3 Learning and Leadership for Healthcare Professionals, Institute of Health, and Care Sciences, Sahlgrenska Academy, Gothenburg University, 41345 Gothenburg, Sweden

**Keywords:** armed conflict, children, disasters, emergencies, human rights

## Abstract

Besides the most common causes of death, children under 14 may suffer from the consequences of disasters and emergencies caused by natural and manmade risks and hazards. These incidents may be prevented by proper risk assessment and the prevention of unnecessary deaths by increasing the safety and well-being of children. An increasing number of manmade incidents are seen in international, non-international, and domestic conflicts when government forces use heavy and light weapons within countries with autocratic leadership. These deaths should be mitigated by holding perpetrators responsible for their deeds and respecting international humanitarian law, human rights, and children’s rights.

## 1. Introduction

Although the United Nations Convention on the Rights of the Child (part one, article one) defines a child as a human being below the age of 18 years [1], some countries such as Singapore legally define a child as someone under the age of 14 [2]; U.S. Immigration Law defines a child as anyone under the age of 21 [3]; and in Islamic Republic of Iran, the legal age for boys and girls are 15 and 13, respectively, which is also the age at which they can marry [4]. However, there seems to be agreement on one applicable age for a child in most countries and that is a human being under 14 years of age, as reported by the world bank [5].

Children up to 14 make up 25% of the world’s population [5]. According to Our World in Data, a platform scientifically maintained by researchers from the University of Oxford to make knowledge on the big problems accessible and understandable, the five most common causes of death among children under 5 are pneumonia, preterm birth, birth asphyxia and trauma, diarrheal diseases, and congenital birth defects [6]. From the ages of 5 to 14, road accidents, cancers, diarrheal diseases, lower respiratory infections, and malaria are the most common causes of death [7]. Besides diseases, an increasing number of natural and man-made hazards endanger children and expose them to new risks as one of the most vulnerable groups in the population, resulting in psychological sequelae, injuries, and deaths [8].

## 2. Disasters and Other Emergencies Caused by Natural Hazards

Disasters caused by natural hazards are expected to affect 175 million children yearly. Flooding, cyclones, drought, heatwaves, storms, and earthquakes are the major natural hazards that affect children’s mental and physical health and learning ability [9]. Natural disasters are associated with an increase of 1.4 infant deaths per 1000 births; however, children are also exposed to man-made hazards, including mass casualty incidents, mass gatherings, mass shootings, and armed conflicts [8,10]. There are some challenging factors in obtaining an accurate number of deaths in diverse disasters, which may particularly influence the outcomes for children and other vulnerable groups. Factors such as risk assessment ability, developing diverse programs for disaster risk management, an ability to determine the necessary resources for a response to disasters or emergencies, the types of preventive and preparedness activities undertaken, and identifying practical and research gaps greatly influence the outcomes [11]. In a report from 2019, the authors presented an estimation of more than 1.5 billion people having been affected because of disasters between 2005 and 2015, including over 700,000 deaths, more than 1.4 million injured people, and around 23 million people made homeless [12]. Therefore, it may be logical to believe that children between the ages of 0–14 have a share of at least 25% in this estimation.

Children are at particular risk in almost all types of disasters due to their specific physiology, anatomy, and behavioral stages, increasing their susceptibility to trauma. In addition, other factors, such as the organization and safety in schools and educational facilities, and pre-existing problems, such as technological dependence or illness, may increase their susceptibility to injuries. Finally, adult behavior may also impact children’s risk of injury and death [8]. These factors facilitate eligible reasons to surge for acute medical services and planning for additional resources to meet the specific needs of children from medical perspectives, i.e., the prevention of all diseases, in particular infectious diseases and other risks and hazards in disasters, including the psychosocial aftermath of the events. One measure as part of such preparedness and preparation is appropriate educational and training initiatives and facilities [13].

## 3. Armed Conflicts-Disasters and Other Emergencies Caused by Manmade Hazards

One of the increasing types of manmade incidents is armed conflicts that particularly impact vulnerable social groups, including children, women, and the elderly. Consequently, reducing its frequency and intensity has been one of the core goals of WHO’s 2030 Sustainable Development Goals [14]. Armed conflicts result in mortality and morbidity; forced displacement; malnutrition; non-fatal physical injuries and disabilities; acute diseases; infectious illnesses; chronic and non-communicable diseases; and mental, sexual, and reproductive health disturbances [15]. According to data compiled by the UN High Commissioner for Refugees (UNHCR), the number of displaced refugees has doubled compared to data from 2008 vs. 2018, reaching 20.4 million. Adding Palestinian refugees under the UN Relief and Work Agency’s protection, the number will increase by another 5.5 million to 25.9 million refugees [15]. Such a huge number of refugees overwhelms the healthcare system in host nations and escalates new outbreaks of infectious diseases if refugees are not distributed equally in larger areas. Previous studies have shown similar mortality rates in three regions of the world: Africa, America, and Asia [16,17]. There is a cumulative total of 6.7–7.5 million infants and 10.1–11.2 million children younger than five years whose deaths could be attributed to conflicts between 1995 and 2015 [17,18]. Firearms and explosives devices directly used in armed conflicts can cause injuries, predominantly in non-combatant civilians, including children and women [16,17,18].

With the development of the hybrid war, there has been a shift from international armed conflicts to non-international conflicts, bringing the battlefield closer to the civilians and exposing the vulnerable population to more risk of displacement, injuries, and deaths. The current war in Ukraine is no exception, with a report of over 7 million refugees and thousands of deaths and injuries [19]. A hybrid war’s main goal is to destroy a country’s infrastructure. As such, it hits medical facilities; energy sections; heat and water resources; and, finally, the financial sector [19]. This is clearly shown in the current Ukrainian–Russian war, whose full impact remains to be discovered. According to the United Nations Human Rights Office of the High Commissioner, there have been 15,246 civilian casualties in Ukraine from 24 February to 3 October 2022. Of these, 6114 were killed, and 9132 were injured. Of a total of 6114 killed, 2380 were men, 1633 were women, 162 were girls, and 193 were boys, as well as 35 children and 1711 adults whose sex is yet unknown. Of the total 9132 injured, 1912 were men, 1382 were women, 196 were girls, 268 were boys, 226 were children, and 5148 were adults whose sex is yet unknown [20].

The transformation of the battlefield to the civilian environment has also facilitated the use of lighter weapons in civilian uprisings and internal conflicts. The use of so-called non-lethal weapons, such as wooden batons or truncheons, rubber or baton rounds, stun grenades, nets, slippery surfaces, and acoustic and electromagnetic pulse weapons, has been shown to cause physical and psychological harm to protesters and even kill them if mishandled. These weapons inflict unnecessary suffering; are used continuously and indiscriminately against children, women, and other vulnerable groups; and cause excessive incidental damage [21,22]. The current uprisings in the Islamic Republic of Iran can be a recent case in which over 400 people have died, including 58 children. About 14,167 persons have been arrested, and their fate is unclear [23,24]. Even in this conflict, like any war, children are impacted by getting killed or maimed, by being recruited or used in paramilitary groups, by armed forces attacking schools and hospitals, by rape or other grave sexual violence, by abduction, and finally by the denial of humanitarian access to children [17,25]. The health consequences of violence are severalfold. One systematic review from India categorized them as mental health, physical health, and behavioral and interpersonal consequences [26]. There were high risks for psychiatric disorders, such as obsessive-compulsive disorders, suicidal behaviors, and depression, as well as increased risks for temperamental problems, poor social adjustment, lack of trust, lower academic performance, and insecure relations with their parents or loved ones. Physical consequences were associated with the type of assaults, and in a few studies, associations between an increased risk of sexually transmitted infections were found [26].

## 4. Preventing Children’s Rights—Recommendations

The outcomes of disasters and emergencies, irrespective of the cause, highlight the importance of preventive measures to avoid any mortality and morbidity in children. The United Nations Convention on the Rights of the Child is a binding agreement signed by countries promising the protection of children’s rights. It explains who children are, all their rights, and the responsibilities of governments. All the rights are connected, equally important, and cannot be taken away from children [27]. Among others, the children’s rights encompass freedom from discrimination; the right to life, survival, and development; freedom of thought and religion; protection from violence; access to health, water, food, and a healthy environment; access to education; protection in war; and many others [27]. However, since there have been continuous violations of children’s rights, necessary measures should be undertaken.

One way to protect children from the brutal consequences of violence and armed conflict is to hold perpetrators, individuals, or nations responsible for implementing and respecting international humanitarian law, human rights, and children’s rights. In December 2012, a bipartisan bill passed by the U.S. Congress was signed into law by President Barack Obama. The act called the Magnitsky intended to punish Russian officials responsible for the death of Russian tax lawyer Sergei Magnitsky in a Moscow prison in 2009 [28]. Later, the Global Magnitsky Act of 2016 authorized the U.S. government to sanction foreign government officials worldwide who are deemed to be human rights offenders, freeze their assets, and ban them from entering the U.S. [29]. Similar laws have been passed and signed in Canada and Europe [30,31]. Such a law should include an official cessation of diplomatic ties and be signed and implemented in other countries to take a real step in preventing the act of violence in children.

Another way is to facilitate International legal proceedings to bring offenders to court, such as the international court. This step, however, requires a resolution made by the United Nations Human Rights Council. Such a resolution was recently issued by the council, leading to an independent investigation into ongoing deadly violence in Iran, which could later pave the way for bringing the responsible perpetrators into the court of justice [32].

Another critical facilitator is to provide a healthcare setting free from political conflicts to provide health services without the risk of arrest, intimidation, and death. As part of this suggestion, the role of the International Committee of Red Cross and Red Crescent should be strengthened and its presence in conflict and wars should be guaranteed [19,32,33].

Finally, besides political actions, children should be educated about their rights and how to handle diverse disasters and emergencies. Scenario-based exercises are an effective educational initiative [13] and should be included in the school curriculum and be trained and exercised at least twice a year. Such exercises may include a simple form of risk assessment and risk management using professional and multiagency staff.

## 5. Conclusions

In conclusion, there is an increasing number of incidents caused by natural and man-made hazards, including various types of armed conflicts, which influence vulnerable populations in society, particularly children. Irrespective of the cause and reasons, children should be seen as the future of our world and should be protected from risk exposure, violence, intimidation, and harm to their lives. Whatever the cause, risk should be mitigated, and responsible perpetrators should be held responsible for their acts, by emphasizing the respect for international humanitarian law, human rights, and children’s rights.

## Data Availability

All included.
